# Supplementary material for: Single-Dose Treatment with Rapamycin Preserves Post-Ischemic Cardiac Function through Attenuation of Fibrosis and Inflammation in Diabetic Rabbit
Source: Int J Mol Sci. 2023 May 19;24(10):8998. doi: 10.3390/ijms24108998 (PMC10218967; doi:10.3390/ijms24108998)
Supplement: Supplementary file 1 [file ijms-24-08998-s001.zip › ijms-2360748-supplementary.pdf]

Supplementary Materials:

Rapamycin Suppressed Phenylephrine-induced Fibroblast Proliferation

Neonatal rat cardiac fibroblast (NRCF) was isolated from 3-4 day-old Wistar rats and digested with collagenase type II (Worthington Biochemical Corp. Lakewood, NJ, USA). Cells were then centrifuged and plated with Dulbecco's modified Eagle's medium (DMEM) supplemented with 10% fetal bovine serum, 100 U/ml penicillin and 100 µg/ml streptomycin. After 1 hour of plating, media were replaced with fresh medium. Cultured NRCF (passage 3) was incubated with or without phenylephrine (PE; 100 µM) or in combination with Rapamycin (RAPA;100 nM) for 48 hours in 96 well plate. Cell proliferation was examined using CellTiter 96 AQueous One Solution Cell Proliferation Assay (Promega Corp. Madison, WI, USA). The cell size was quantified using Image J software (NIH, Bethesda, MD). Treatment with PE (100 µM for 48 hours) stimulated NRCF proliferation, which was inhibited by co-treatment with RAPA (100 nM) with reduction of cell size (Supplement Figure S1 A,B).

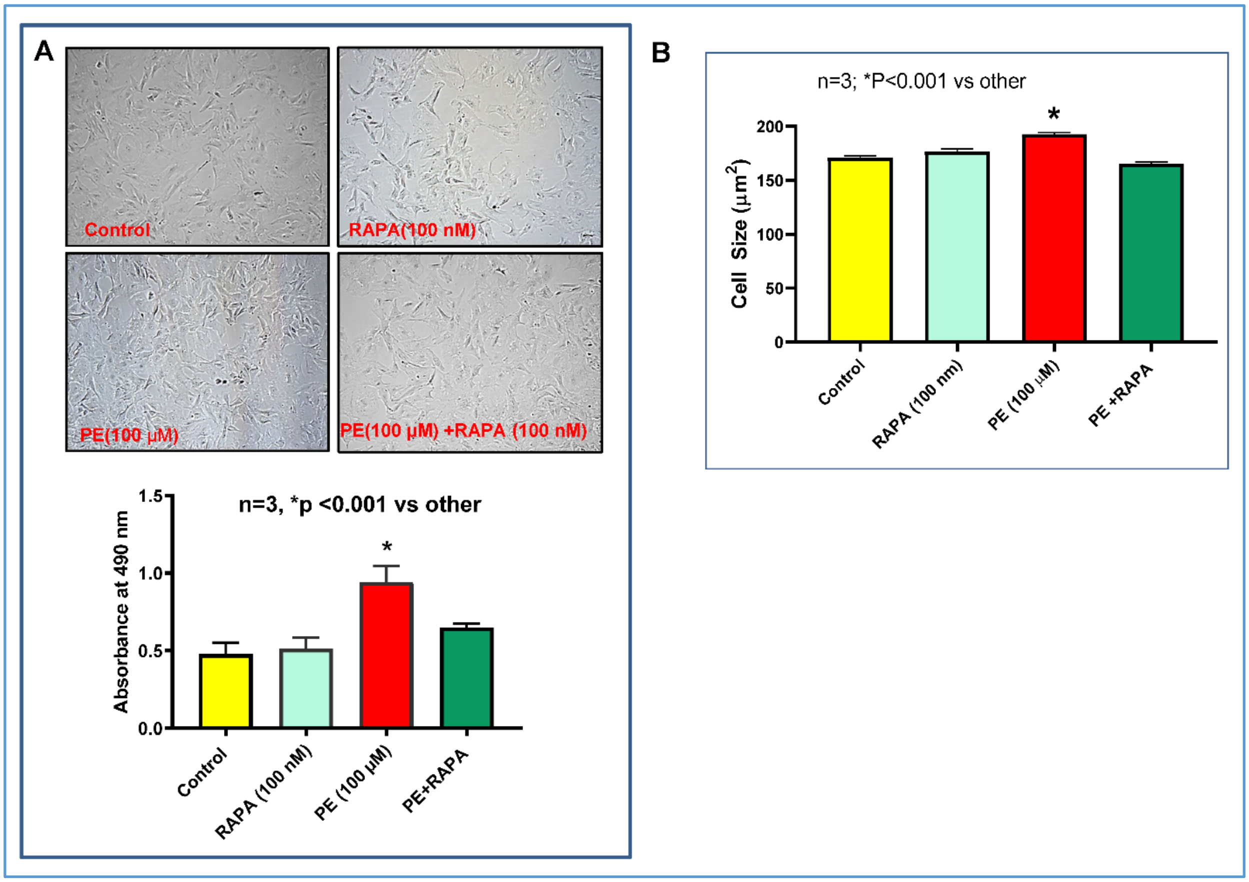

Figure S1: A. Representative pictures of neonatal rat cardiac fibroblast (NRCF) after 48 hours of treatment with phenylephrine (PE; 100 µM) or in combination with Rapamycin (RAPA;100 nM). Lower Panel shows the cell proliferation. **B.** Cell size after treatment with PE and/or RAPA.

Rapamycin Suppressed Phenylephrine-induced Cardiomyocyte Hypertrophy under Hyperglycemic Condition

Human ventricular cardiomyocytes (AC-16; Sigma-Aldrich, Inc; St. Louis, MO 68178, United States) were incubated with high glucose (HG: 33 mM) for 72 hours [1]. Then cells were treated with or without phenylephrine (PE; 100 µM) or in combination with Rapamycin (RAPA;100 nM) for 48 hours. Cells were fixed with 4% paraformaldehyde and then stained with WGA (Wheat germ agglutinin, Lectin from triticum vulgaris conjugated with FITC, cat#L4895, Sigma-Aldrich, St. Louis, MO, USA) for 30 minutes. The slides were then washed three times in PBS and mounted using ProLong™ Diamond Antifade Mounting with DAPI (Cat # P36966, ThermoFisher Scientific, Waltham, MA). The cell size was quantified using Image J software (NIH, Bethesda, MD). After taking pictures under a confocal microscope (Nikon D-Eclipse C1 confocal microscope), the cell size was quantified using Image J software (NIH, Bethesda, MD). Our data showed that cardiomyocyte size was significantly increased after PE treatment under hyperglycemic condition as compared to control (HG treated cells). RAPA abolished the hypertrophic effect of PE in cardiomyocytes under hyperglycemic condition (**Supplementary Figure S2).** RAPA treatment alone also reduced the cell size as compared to HG-control.

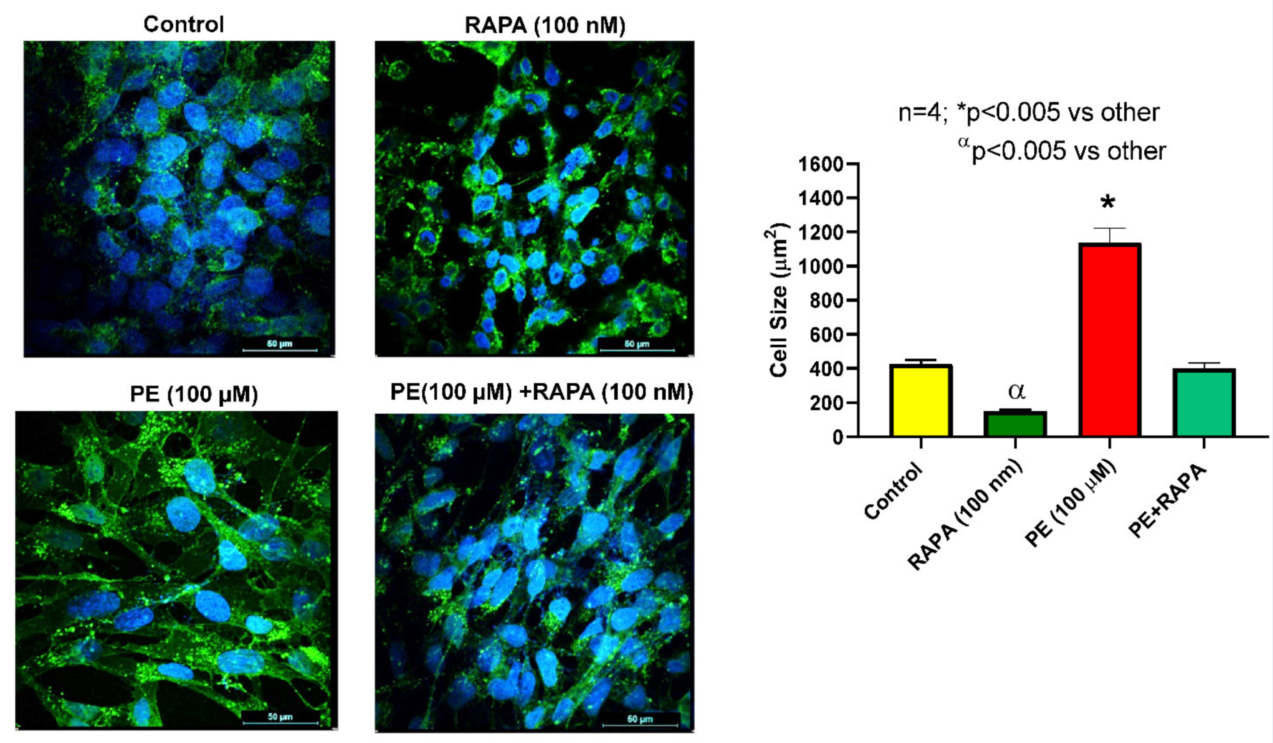

Figure S2: Left Panel: Representative pictures of the FITC-labeled WGA (FITC-WGA) staining of AC-16 cells after 48 hours of treatment with phenylephrine (PE; 100 µM) or in combination with Rapamycin (RAPA; 100 nM) under hyperglycemic condition (33 mM glucose). **Right Panel** shows the quantification of average cell size after staining with WGA.

Reference:

1. Samidurai, A., Ockaili, R., Cain, C., Roh, S. K., Filippone, S. M., Kraskauskas, D., Kukreja, R. C. & Das, A. (2020) Differential Regulation of mTOR Complexes with miR-302a Attenuates Myocardial Reperfusion Injury in Diabetes, iScience. 23, 101863.
